# Beyond BMI: The Clinical Relevance of Sarcopenia and Muscle Quality in Gynecologic Oncology from Meta-Analyses to Practice—The Potential Role of Body Composition Assessment, Including BIA, in Supportive Care

**DOI:** 10.3390/cancers18193164

**Published:** 2026-10-01

**Authors:** Beata Pięta, Karolina Gajewska, Małgorzata Mikołajczyk, Michalina Wiśniewska, Alicja Rzymska, Bartłomiej Burzyński, Paweł Rzymski

**Affiliations:** 1 Department of Practical Midwifery Education, Poznan University of Medical Sciences, 60-781 Poznan, Poland; 2 Department of Cardiology, Poznan University of Medical Sciences, 61-848 Poznan, Poland; 3 Doctoral School, Poznan University of Medical Sciences, 60-812 Poznan, Poland; 4 Students’ Scientific Society, Poznan University of Medical Sciences, 60-781 Poznan, Poland; 5 Department of Rehabilitation, Faculty of Health Sciences in Katowice, Medical University of Silesia in Katowice, 40-635 Katowice, Poland; 6 Department of Mother’s and Child’s Health, Gynecologic and Obstetrical University Hospital, Poznan University of Medical Sciences, Polna St 33, 60-535 Poznan, Poland

**Keywords:** sarcopenia, low muscle mass, muscle radiodensity, body composition, gynecological malignancies, treatment toxicity, bioelectrical impedance analysis, quality of life

## Abstract

Sarcopenia, low muscle mass, and low muscle radiodensity are increasingly being considered factors associated with treatment outcomes in patients with gynecological malignancies. Despite the growing body of evidence, body composition assessment is not routinely incorporated into clinical practice. The aim of this review was to present current evidence on the associations of muscle parameters with survival, complications, systemic treatment toxicity, and quality of life, as well as to discuss the opportunities and limitations of computed tomography (CT) and bioelectrical impedance analysis (BIA) in body composition monitoring. A narrative literature review was conducted using the PubMed, Scopus, and Web of Science databases, primarily covering publications from 2019 to 2026, together with earlier studies relevant to the topic. Systematic reviews, meta-analyses, and selected observational, longitudinal, and interventional studies were included. The available evidence indicates that low muscle mass and low muscle radiodensity are primarily associated with poorer overall survival and an increased risk of selected postoperative complications. Evidence regarding progression-free survival, disease recurrence, chemotherapy toxicity, and quality of life remains less consistent. Low muscle mass may occur independently of body weight and body mass index (BMI); therefore, routine anthropometric assessment may fail to identify adverse changes in body composition. CT image analysis enables quantitative assessment of muscle mass and evaluation of muscle radiodensity, whereas BIA may serve as a complementary tool for repeated body composition monitoring. However, its use is limited by the effects of hydration status, edema, and ascites, as well as by the lack of validated cut-off values for patients with gynecological malignancies. Body composition assessment should be interpreted in conjunction with evaluations of muscle strength, physical performance, nutritional status, treatment toxicity, and quality of life. Prospective studies are needed to determine whether body composition monitoring and the implementation of targeted supportive care improve treatment tolerance and clinical outcomes.

## Simple Summary

Changes in body composition, particularly loss of muscle mass and reduced muscle quality, may affect treatment outcomes in patients with gynecological cancers. However, these changes are not routinely assessed in clinical practice and may remain undetected when body weight or body mass index is used alone. This review aims to summarize current knowledge on the relationships between muscle characteristics and survival, postoperative complications, treatment-related toxicity, and quality of life. It also discusses the potential use of computed tomography and bioelectrical impedance analysis for monitoring body composition. The findings suggest that low muscle mass and reduced muscle quality may be associated with poorer survival and selected treatment complications. This review highlights the importance of incorporating body composition assessment into comprehensive patient care and identifies areas requiring further research. Future prospective studies should determine whether regular monitoring and targeted supportive interventions can improve treatment tolerance and clinical outcomes.

## 1. Introduction

Advances in the diagnosis and treatment of gynecological malignancies have contributed to improvements in clinical outcomes. Nevertheless, many patients continue to experience postoperative complications, systemic treatment toxicity, deterioration in physical functioning, and reduced quality of life. In recent years, increasing attention has been paid to body composition as a potential factor associated with disease course, treatment tolerance, and clinical outcomes [1,2,3]. Particular interest has focused on skeletal muscle mass and quality, which provide information that is not captured by routine assessments of body weight and body mass index (BMI).

Sarcopenia is a progressive and generalized skeletal muscle disorder associated with an increased risk of adverse clinical outcomes. According to the contemporary approach, low muscle strength is the primary criterion for identifying probable sarcopenia, whereas low muscle quantity or quality is used to confirm the diagnosis. In severe cases, impaired physical performance is also present [4]. Sarcopenia was originally described in the context of aging, but it may also develop as a secondary condition in the course of chronic diseases, including malignant neoplasms [1,4]. Its development in oncology populations is multifactorial and may be associated with chronic inflammation, metabolic disturbances, inadequate energy and protein intake, reduced physical activity, advanced disease, and the consequences of anticancer treatment [1].

In many oncology studies, however, the term “sarcopenia” has been used solely on the basis of muscle mass assessment, without simultaneous measurement of muscle strength and physical performance. The most commonly used parameter has been the skeletal muscle index (SMI), calculated from cross-sectional CT images at the level of the third lumbar vertebra (L3) [2,3]. CT also enables the assessment of muscle attenuation, which is an indirect imaging marker of muscle radiodensity and intramuscular lipid content [3,5]. Therefore, in studies using imaging parameters alone, the terms “low muscle mass” and “low muscle attenuation” are more precise than equating these findings with a complete clinical diagnosis of sarcopenia.

Analysis of CT images obtained during routine oncological assessment enables the evaluation of body composition without exposing patients to additional radiation. Its broader use is limited by the need for appropriate software, the time required for tissue segmentation, and the lack of uniform cut-off values. Consequently, simpler methods that enable repeated monitoring of body composition changes are being explored. One such method is bioelectrical impedance analysis (BIA), which may serve as a complementary tool but does not replace CT assessment or the functional criteria used to diagnose sarcopenia. Low muscle mass can be assessed using several complementary methods. In oncological research, CT analysis at the third lumbar vertebral level is most frequently used to calculate skeletal muscle area and the height-normalized skeletal muscle index, while CT-derived muscle attenuation provides additional information on radiological muscle quality. Dual-energy X-ray absorptiometry and BIA estimate whole-body or appendicular lean tissue, although BIA results are more sensitive to hydration disturbances, edema, and ascites. Because muscle quantity does not directly reflect muscle function, imaging and body-composition measurements should be interpreted together with muscle-strength and physical-performance assessments when clinical sarcopenia is considered.

Low muscle mass may occur independently of body weight and BMI, including in patients who are overweight or obese. This condition, referred to as sarcopenic obesity, may remain undetected if patient assessment is limited to body weight and BMI [1,6]. This may be relevant to systemic treatment because the doses of many cytotoxic agents are based on body surface area, which does not directly reflect the amount of lean body mass. A higher drug dose per kilogram of lean body mass may be one of the mechanisms contributing to greater susceptibility to adverse effects among patients with low muscle mass. The available evidence more strongly supports the prognostic significance of adverse muscle parameters than their predictive value. Therefore, their use in therapeutic decision-making requires further investigation.

Body composition assessment may complement a comprehensive clinical evaluation that includes nutritional status, muscle strength and physical performance, treatment toxicity, and quality of life. Body composition results should not constitute an independent basis for modifying therapy, but they may support the identification of patients requiring more detailed assessment and individually tailored nutritional, physiotherapeutic, and supportive care. The implementation of such an approach requires collaboration within an interdisciplinary team that includes physicians, dietitians, physiotherapists, psycho-oncologists, oncology nurses, and midwives.

## 2. Aim of the Review

The aim of this structured narrative review is to present current evidence on the associations of sarcopenia, low muscle mass, and low muscle attenuation with survival, complications, treatment toxicity, and quality of life in patients with gynecological malignancies. An additional aim is to discuss the opportunities and limitations of CT and BIA in body composition assessment, the potential role of monitoring these parameters in supportive care, and the principal directions for further research.

## 3. Literature Search Methods

**Figure 1:**
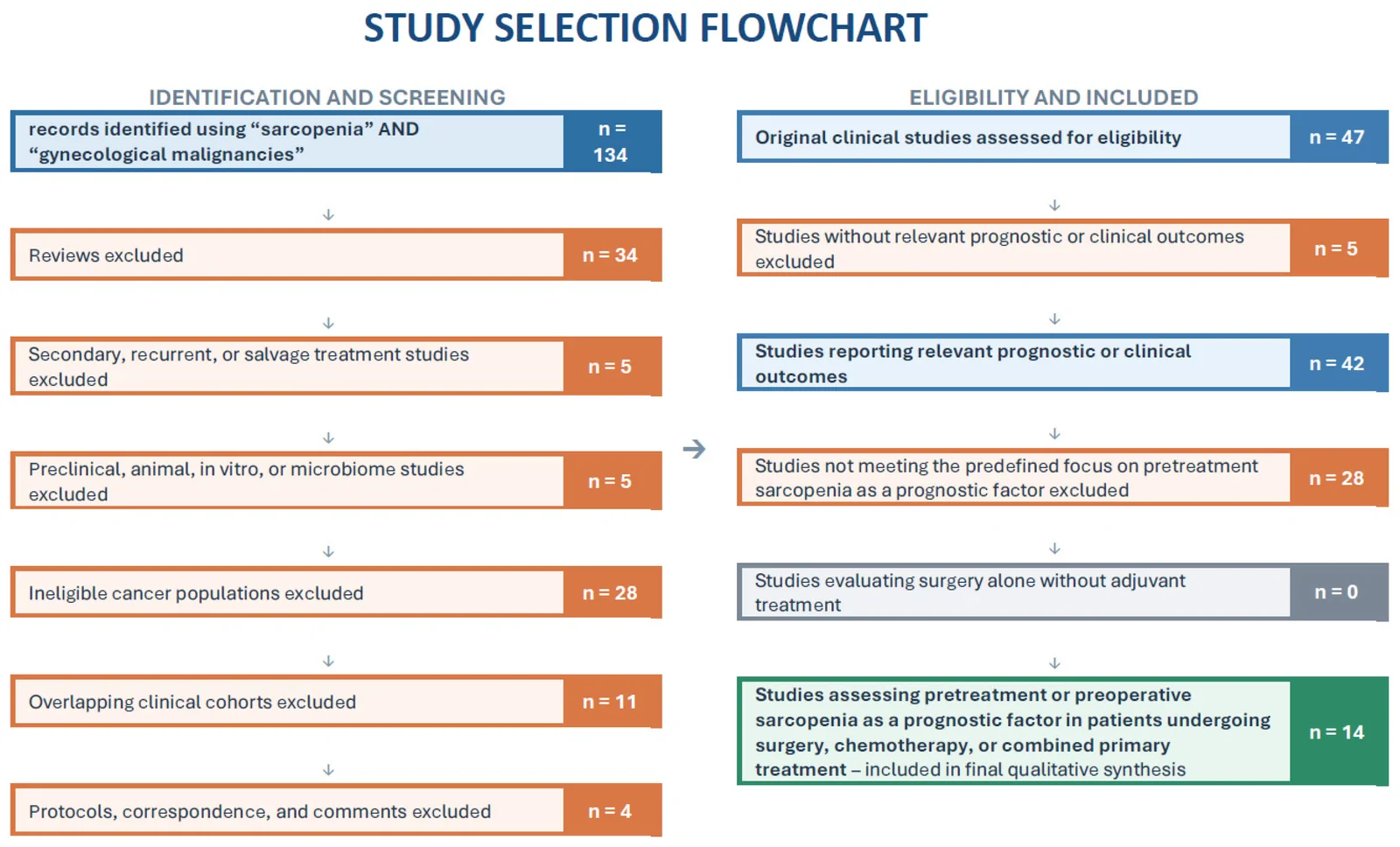
Flowchart of Study Selection for the Prognostic Assessment of Pretreatment Sarcopenia in Gynecological Malignancies.

A structured narrative review of the literature concerning the significance of body composition, muscle mass and quality, and sarcopenia in patients with gynecological malignancies was conducted. PubMed/MEDLINE, Scopus, and Web of Science were searched for publications available up to August 2026. The database search using the terms “sarcopenia” AND “gynecological malignancies” identified 134 records. Following title, abstract, and publication-type screening, 34 reviews, systematic reviews, and meta-analyses were excluded. Five studies performed exclusively in secondary, recurrent, or salvage treatment settings, five preclinical, animal, in vitro, or microbiome studies, and 28 studies involving ineligible cancer populations were also excluded. The ineligible population category included studies concerning malignancies other than ovarian, endometrial, or cervical cancer, mixed cancer populations without separately extractable data, and non-malignant conditions. A further 11 publications were excluded because they were considered to represent overlapping clinical cohorts. Cohort overlap was suspected when publications originated from the same institution, involved the same or a substantially similar author group, covered overlapping recruitment periods, and included comparable patient and treatment characteristics. In such cases, the most representative publication was retained. Four additional records were excluded because they were protocols, correspondence articles, comments, or other publications without original clinical results. A total of 47 original clinical studies were retained for detailed assessment. Five studies were subsequently excluded because they did not report relevant prognostic or clinical outcomes, including treatment response, survival, recurrence or progression, mortality, treatment-related toxicity, perioperative or postoperative complications, or length of hospital stay. Thus, 42 studies reporting at least one relevant clinical outcome were evaluated according to the treatment setting and timing of sarcopenia assessment. Of these, 14 studies provided robust pretreatment or preoperative data on sarcopenia, skeletal muscle mass, or muscle quality as potential prognostic factors in patients undergoing surgery, chemotherapy, or combined primary treatment. Studies with longitudinal muscle assessment were included when they also contained a relevant pretreatment or preoperative measurement. The remaining 28 studies did not meet this specific final eligibility criterion and were excluded. Ultimately, 14 studies were included in the qualitative synthesis and presented in the summary table. The study selection process is illustrated in Figure 1. Only full-text articles published in English were eligible for the final qualitative synthesis if they: (1) included adult patients with ovarian, endometrial, or cervical cancer; (2) evaluated sarcopenia, skeletal muscle mass, skeletal muscle index, muscle attenuation, muscle radiodensity, muscle quality, or another imaging-derived muscle parameter before treatment or before surgery; (3) reported at least one relevant clinical outcome, including treatment response, survival, recurrence or progression, mortality, treatment-related toxicity, postoperative complications, or length of hospital stay; and (4) provided original clinical data from a non-overlapping patient cohort. Longitudinal studies evaluating muscle loss during treatment were eligible only if they also included a relevant pretreatment or preoperative muscle assessment. Studies examining the use of BIA and nutritional, exercise-based, and prehabilitation interventions were analyzed separately. Owing to the heterogeneity of populations, definitions of sarcopenia, measurement methods, and study outcomes, no quantitative synthesis of the results was performed.

## 4. Sarcopenia, Survival, and Complications in Gynecological Malignancies

The available evidence consistently indicates that sarcopenia and adverse body composition parameters are associated with poorer prognosis among patients with gynecological malignancies. These associations relate primarily to overall survival and the risk of treatment-related complications, whereas findings concerning progression-free survival and the risk of disease recurrence remain less conclusive. Most evidence is derived from studies involving patients with ovarian cancer, although similar associations have also been observed in endometrial and cervical cancer. The strength and clinical context of the available evidence differ substantially across gynecological cancer types. Ovarian cancer has the largest evidence base, particularly for the associations of CT-derived low muscle mass and attenuation with overall survival and postoperative complications. Evidence in cervical cancer is less extensive and is derived predominantly from patients receiving definitive radiotherapy or concurrent chemoradiotherapy, with several studies focusing on treatment-associated muscle loss rather than baseline SMI alone. Data in endometrial cancer remain more limited and heterogeneous, covering surgical outcomes, adjuvant treatment tolerance, chemotherapy-induced toxicity, and, more recently, immunotherapy; therefore, findings from ovarian cancer should not be generalized to endometrial or cervical cancer without tumor-specific confirmation.

When interpreting the findings, the heterogeneity of the terminology used should be taken into account. A clinical diagnosis of sarcopenia includes an assessment of muscle strength and muscle quantity or quality and, in severe cases, physical performance. However, in many oncology studies, the term “sarcopenia” has been applied on the basis of a reduced skeletal muscle index assessed by CT, without a simultaneous evaluation of muscle strength and physical performance. In such cases, the findings primarily refer to imaging-defined low muscle mass, which should be acknowledged in their interpretation. Throughout this review, clinical sarcopenia, imaging-defined low muscle mass, muscle loss, and poor muscle quality are treated as distinct but related constructs. The term “clinical sarcopenia” refers to a diagnosis incorporating low muscle strength together with reduced muscle quantity or quality, while poor physical performance indicates severe sarcopenia. In contrast, low SMI denotes imaging-defined low muscle quantity; reduced CT muscle attenuation or radiodensity is considered an indirect marker of myosteatosis and impaired muscle quality; and muscle loss describes a longitudinal reduction between repeated measurements. When the original studies used “sarcopenia” on the basis of CT-derived SMI alone, this terminology is reported for consistency with the source publication but interpreted in the present review as imaging-defined low muscle mass [4].

In the meta-analysis by Lin et al., which included 16 studies and 2031 patients with gynecological malignancies, sarcopenia was associated with a higher risk of death, reflected by overall survival (OS; HR 2.61; 95% CI: 1.52–4.46), and a higher risk of disease progression, reflected by progression-free survival (PFS; HR 1.37; 95% CI: 1.09–1.73) [3]. These associations were observed despite heterogeneity in the definitions of sarcopenia and in the SMI cut-off values applied.

The magnitude of the observed associations varied according to cancer type and study population characteristics. In the study by Bronger et al., which included patients with advanced high-grade serous ovarian cancer, low muscle mass at baseline was associated with a higher risk of death (HR 2.89; 95% CI: 1.11–7.54) [7]. Among patients with locally advanced cervical cancer, Lee et al. demonstrated that an SMI loss exceeding 10% per 150 days during chemoradiotherapy was associated with an increased risk of death (HR 6.02; 95% CI: 3.04–11.93) [8]. In the study by de Paula et al., low SMI in patients with endometrial cancer was associated with poorer one-year survival (HR 2.23; 95% CI: 1.19–4.20) [9]. The wide confidence intervals in some analyses indicate limited precision and support a cautious approach when comparing effect sizes across different cancer types.

The association between low muscle mass and poorer prognosis may have a complex basis. In addition to its role in movement and the maintenance of physical function, muscle tissue constitutes an amino acid reserve used in metabolic, immune, and regenerative processes. A reduction in this reserve may impair the body’s capacity to respond to the stress associated with malignant disease, extensive surgery, and systemic therapy. Low muscle mass may also coexist with chronic inflammation, malnutrition, reduced physical activity, and advanced disease. These associations, however, do not establish a direct causal relationship between sarcopenia and poorer treatment outcomes.

The possibility that low muscle mass may occur independently of BMI is also clinically important. Normal or elevated BMI does not exclude muscle tissue deficiency, including sarcopenic obesity. Limiting patient assessment to body weight and BMI may therefore result in failure to identify adverse body composition changes and the actual clinical risk.

More detailed evidence concerning ovarian cancer was provided by the meta-analysis by Ge et al., which included 22 studies [5]. Low SMI was associated with an approximately 30% increase in the overall risk of death (HR 1.30; 95% CI: 1.07–1.58). Low muscle quality, assessed on the basis of reduced muscle attenuation on CT, was also associated with poorer survival (HR 1.24; 95% CI: 1.03–1.49) and an increased risk of complications, particularly postoperative complications. In patients with low muscle attenuation, the relative risk of complications was almost twice as high (RR 1.99; 95% CI: 1.04–3.79). Low SMI was also associated with a higher risk of complications (RR 1.49; 95% CI: 1.13–1.98). These findings suggest that the prognostic value of body composition assessment may extend beyond muscle quantity to include muscle quality.

In the same meta-analysis, no significant association was confirmed between low SMI or low muscle attenuation and PFS. The HR was 1.15 for low SMI (95% CI: 0.95–1.40) and 1.22 for low muscle attenuation (95% CI: 0.69–2.16). No significant association was found between low SMI and the risk of disease recurrence (HR 1.08; 95% CI: 0.36–1.37) [5]. Thus, the association of adverse muscle parameters with overall survival appears to be more consistent than their association with disease progression or recurrence. Discrepancies among meta-analyses may result from differences in patient characteristics, cancer type, disease stage, treatment approach, and the methods used to define low muscle mass and quality.

An earlier systematic review and meta-analysis by Ubachs et al. also demonstrated an adverse association between low muscle mass and quality and survival among patients with ovarian cancer [2]. In that analysis, low SMI was associated with an approximately 11% increase in the risk of death (HR 1.11; 95% CI: 1.03–1.20), whereas low muscle attenuation was associated with an approximately 14% increase in this risk (HR 1.14; 95% CI: 1.08–1.20) [2]. These findings should be interpreted as relative increases in mortality risk, rather than percentage reductions in survival time. The authors also emphasized the considerable variation in SMI and skeletal muscle radiodensity or attenuation cut-off values, as well as the limited methodological quality of some of the included studies. Despite these limitations, the direction of the observed associations remained relatively consistent.

Sarcopenia and low muscle mass should be distinguished from cancer cachexia. These conditions are related but not synonymous. Loss of muscle mass and function may be an important component of cachexia, but it may also occur independently as a result of aging, inadequate nutrient intake, reduced physical activity, comorbidities, or treatment toxicity. Therefore, muscle assessment should form part of a broader evaluation of nutritional status, physical performance, and disease severity.

From the perspective of supportive care, evidence concerning postoperative complications and the course of hospitalization is particularly important. Meta-analyses and cohort studies indicate that patients with low muscle mass or quality are generally at greater risk of infectious complications, prolonged hospitalization, delays in subsequent treatment, and poorer treatment tolerance.

In the study by Mercan et al., patients with low muscle mass had a higher risk of infectious complications (RR 3.16; 95% CI: 1.21–8.28) [10]. Van der Zanden et al. demonstrated that both low SMI (RR 2.00; 95% CI: 1.06–3.77) and low muscle attenuation (RR 2.18; 95% CI: 1.26–3.76) were associated with a higher risk of postoperative infections [11]. These findings suggest that the risk of complications is associated with both muscle quantity and muscle quality.

Adverse muscle parameters may also be associated with a prolonged hospital stay. In the study by Chae et al., the length of hospitalization was 17 days among patients with sarcopenia and 12 days among those with normal muscle mass [12]. In the study by Rutten et al., the corresponding lengths of stay were 15.3 and 13.6 days [13]. Although these differences indicate a potentially greater burden of perioperative care, their clinical significance should be interpreted in the context of surgical extent, disease stage, patient age, comorbidities, and other factors that may influence the length of hospitalization.

An increased incidence of complications and prolonged hospitalization may delay subsequent treatment, necessitate treatment modifications, and increase the burden of perioperative care. However, the studies discussed do not allow a definitive conclusion that low muscle mass is a direct cause of all observed complications. Low muscle mass may also reflect advanced disease, malnutrition, chronic inflammation, older age, or coexisting functional impairment.

The meta-analysis by Allanson et al. [14], which included 13 studies and 2446 patients, demonstrated an association between sarcopenia and poorer prognosis in gynecological malignancies. Sarcopenia was associated with a higher risk of death (OS; HR 1.33; 95% CI: 1.08–1.64) and disease progression (PFS; HR 1.69; 95% CI: 1.03–2.76) [14]. No statistically significant association was observed with treatment-related adverse events (HR 1.28; 95% CI: 0.69–2.40). Treatment tolerance could not be evaluated because none of the studies included in the meta-analysis reported this endpoint.

Di Fiore et al. emphasized that the prevalence of sarcopenia in gynecological malignancies may range from approximately 20% to 70%, depending on cancer type, population characteristics, assessment method, and the cut-off values adopted [15]. This broad range reflects substantial methodological variation and confirms the need to standardize the assessment of muscle mass, quality, and function.

The findings presented support the inclusion of body composition assessment in the comprehensive evaluation of patients with gynecological malignancies. Body weight and BMI alone do not enable the reliable identification of low muscle mass or the assessment of muscle quality. At the same time, CT-derived parameters should not be interpreted separately from muscle strength, physical performance, nutritional status, inflammatory burden, and the clinical course of the disease.

The identification of patients with low muscle mass or quality provide a basis for more detailed clinical evaluation and referral for individually tailored supportive interventions. Particular attention should be paid to combining nutritional support with physical activity, especially resistance exercise, because nutritional intervention alone are usually insufficient to preserve or restore muscle function. During the perioperative period, these measures may be delivered as part of multimodal prehabilitation and care consistent with Enhanced Recovery After Surgery protocols. The scope of the intervention, including energy and protein intake, should be tailored to nutritional status, metabolic requirements, organ function, and the current stage of treatment. Among patients requiring increased protein intake, a range of 1.2–1.5 g/kg body weight per day may be considered [1].

## 5. Chemotherapy, Muscle Loss, and Treatment Toxicity

Chemotherapy may contribute to accelerated muscle loss in patients with cancer. Longitudinal CT-based studies have shown that systemic treatment may be accompanied by a significant reduction in muscle mass that is not always reflected by changes in total body weight [16,17]. This phenomenon is clinically relevant because the doses of many cytotoxic agents are determined on the basis of body surface area without direct consideration of lean body mass. Consequently, patients with low muscle mass may receive a higher drug dose per kilogram of lean body mass, potentially increasing drug exposure and the risk of adverse effects [18].

The available evidence suggests that low muscle mass may be associated with a greater risk of chemotherapy toxicity. In studies involving patients with colorectal and breast cancer, low baseline muscle mass was associated with more frequent adverse events, hospitalizations, and toxicity leading to treatment modifications [18,19,20]. These findings suggest that adverse muscle parameters may have clinical significance. However, they are derived from studies involving other cancer types, and their relevance to patients with gynecological malignancies requires confirmation.

Dynamic muscle loss during treatment may also have prognostic significance. An association between muscle loss and poorer survival has been observed in patients with ovarian cancer receiving neoadjuvant chemotherapy and in patients receiving systemic treatment for upper gastrointestinal malignancies [16,17]. Owing to the observational nature of these studies, however, the findings do not establish a direct causal relationship.

The association between body composition and the risk of chemotherapy-induced peripheral neuropathy (CIPN) is of particular interest, especially during paclitaxel therapy. Evidence suggests that the drug dose relative to lean body mass is clinically relevant. In a retrospective study by Aichi et al. involving 98 patients with endometrial cancer treated with carboplatin and paclitaxel, the paclitaxel dose per kilogram of LBM was independently associated with CIPN (OR 2.58; 95% CI: 1.08–6.19) [21]. The cut-off value was 8.12 mg/kg LBM. Neuropathy occurred in 52.0% of patients receiving a dose at or above this value and in 30.1% of those with lower exposure (*p* = 0.049) [21]. These findings indicate that the paclitaxel dose relative to lean body mass is a potential indicator of CIPN risk. However, further validation is required before this measure can be used to guide dosing decisions.

From the perspective of midwifery practice, systematic observation of patients receiving treatment for gynecological malignancies and identification of symptoms that may indicate neurotoxicity may be important. These symptoms include numbness, paresthesia, neuropathic pain, sensory disturbances, balance impairment, and reduced manual dexterity. Early reporting of such symptoms to the treatment team enables more detailed assessment and consideration of appropriate management. Because studies directly evaluating the role of midwives in the detection of CIPN are lacking, these activities should be presented as elements of supportive care and interdisciplinary collaboration, rather than interventions with proven effectiveness in preventing permanent complications.

Analysis of CT images obtained routinely for oncological diagnosis and treatment monitoring may enable the assessment of muscle mass changes occurring during chemotherapy [16,17]. Despite limitations related to the effects of hydration status, edema, and ascites, BIA is used as a complementary and simple tool for body composition monitoring in clinical practice [22]. Combining body composition assessment with evaluations of nutritional status, functional performance, and clinical symptoms may facilitate the identification of patients requiring nutritional, physiotherapeutic, or other supportive interventions. The effect of this approach on treatment tolerance and quality of life requires confirmation in prospective studies. More recent evidence from gynecological malignancies supports the clinical relevance of combining pretreatment assessment with longitudinal monitoring of skeletal muscle. In patients with ovarian cancer undergoing primary debulking surgery followed by platinum-based chemotherapy, Lee et al. found that a low pretreatment prognostic nutritional index was associated with subsequent skeletal muscle loss, while muscle loss independently predicted mortality (HR 2.27; *p* < 0.001). Similarly, in patients with endometrial cancer treated with immune checkpoint inhibitors, Katayama et al. reported that a decrease in SMI between baseline assessment before cancer-directed therapy and ICI initiation was associated with poorer overall survival (HR 3.64; 95% CI: 1.11–11.94). Although limited by their retrospective design, these findings suggest that assessment of both pretreatment muscle status and its subsequent trajectory may improve prognostic stratification in patients with gynecological malignancies.

Key studies in gynecological oncology supporting the associations described above are summarized in Table 1.

## 6. Quality of Life, Sarcopenia, and Changes in Body Composition

Health-related quality of life (HRQoL) is one of the key endpoints in the treatment of gynecological malignancies, alongside overall survival, progression-free survival, treatment toxicity, and symptom control. It is particularly important among patients undergoing prolonged systemic therapy and among those with recurrent or advanced disease, for whom maintaining function, independence, and the ability to perform everyday activities may be a major goal of care.

Quality of life is a multidimensional concept encompassing physical, emotional, cognitive, and social functioning, as well as the burden of symptoms associated with the disease and its treatment. It should not be equated solely with performance status or survival. Poorer physical functioning and shorter survival may coexist with reduced quality of life, but they are not synonymous with a direct deterioration in patient-reported outcomes.

Sarcopenia, low muscle mass, and poor muscle quality may affect daily functioning by reducing strength and mobility, increasing fatigue, and limiting the ability to perform ordinary activities. The studies discussed previously demonstrated associations between adverse muscle parameters and poorer survival and a greater risk of complications in gynecological oncology [2,15]. This does not, however, mean that the association between body composition and quality of life has been documented equally well in this population.

Most studies of sarcopenia in patients with gynecological malignancies have focused on OS, PFS, postoperative complications, and treatment toxicity. Quality of life has been assessed less frequently and has not always been measured using standardized instruments. Therefore, evidence concerning the direct relationship of SMI and muscle attenuation with HRQoL in gynecological oncology remains limited. Particular caution is necessary when extrapolating findings from patients with other malignancies to those with gynecological cancers.

Indirect evidence concerning the association between nutritional status and quality of life was provided by the study by Daly et al. among patients with incurable cancer [25]. HRQoL was assessed using the EORTC QLQ-C30 questionnaire, which includes global health status, physical functioning, role functioning, emotional, cognitive, and social functioning, as well as the severity of major symptoms. A higher grade of weight loss, classified using a system that incorporates baseline BMI and the percentage of weight lost, was associated with poorer quality of life [25].

Patients classified in the higher weight-loss grades had poorer global quality of life, physical functioning, and role functioning, and reported greater fatigue, pain, appetite loss, and limitations in daily activities [25]. These findings indicate that deterioration in nutritional status may coexist with a greater symptom burden and functional limitations. However, the study concerned weight loss rather than sarcopenia or SMI changes specifically. Therefore, weight loss cannot be equated with muscle tissue loss on the basis of these findings.

Higher grades of weight loss were also associated with poorer survival, and the risk of death was almost twice as high in the highest category [25]. This finding concerns prognosis rather than quality of life directly. The two outcomes should be analyzed separately, although deterioration in nutritional status may adversely affect both survival and daily functioning.

Longitudinal studies have shown that muscle mass may decrease during chemotherapy without a simultaneous reduction in total body weight [17]. This means that routine weighing may fail to identify progressive muscle tissue loss. However, there is insufficient evidence to conclude that a decrease in SMI invariably precedes a clinically meaningful deterioration in quality of life. This is a plausible hypothesis, but it requires confirmation in prospective studies in which body composition and HRQoL are assessed simultaneously at successive stages of treatment.

Cancer-related fatigue is an important symptom linking nutritional status, physical performance, and quality of life. It may limit activity, impair occupational and social functioning, and reduce independence. The review by Inglis et al. suggests that selected nutritional interventions may reduce fatigue in some patients with cancer [26]. However, the individual studies were heterogeneous in terms of population, type and duration of the intervention, and the assessment instruments used.

On the basis of these findings, it cannot be concluded that nutritional intervention alone restores muscle mass or improves quality of life in all patients with gynecological malignancies. Nutritional support should be tailored to individual needs and, whenever permitted by the patient’s clinical condition, combined with appropriately selected physical activity and treatment of symptoms that limit food intake or mobility [1,26].

The association between body composition and quality of life should be considered in the context of multiple coexisting factors. HRQoL scores may be influenced by cancer stage and site, treatment type, pain severity, fatigue, nausea, neuropathy, nutritional disturbances, ascites, the presence of a stoma, comorbidities, psychological status, and social support. Low muscle mass may be one component of this complex clinical picture, but it should not be considered the sole cause of reduced quality of life.

Body composition monitoring should therefore be complemented by functional assessment and patient-reported outcomes. The use of standardized instruments, such as the EORTC QLQ-C30 and cancer-specific modules, enables the assessment of changes during treatment and the identification of areas requiring intervention. Simultaneous assessment of body composition, muscle strength, physical performance, nutritional status, and HRQoL provide a more comprehensive picture of the patient’s condition than the analysis of a single parameter.

Oncology nurses and midwives, in accordance with the organization of care and their professional competencies, may play an important role in the regular monitoring of symptoms and functional changes. Particular attention should be paid to increasing fatigue, reduced activity, difficulty walking, loss of independence, reduced appetite, unintentional weight loss, and poorer treatment tolerance. The identification of such changes should prompt a more detailed evaluation and, depending on the patient’s needs, referral for medical, dietary, physiotherapeutic, psycho-oncological, or pain management consultation.

There is currently insufficient evidence to conclude that regular body composition measurements alone improve quality of life. Nevertheless, these measurements may support the early identification of adverse trends and facilitate referral for appropriate supportive care. The clinical significance lies not in the measurement itself, but in its interpretation in conjunction with symptoms, nutritional status, muscle strength and physical performance, and quality-of-life questionnaire results.

The integration of body composition monitoring with regular HRQoL assessment may represent a valuable component of holistic care for patients with gynecological malignancies. However, prospective studies are needed to determine whether changes in SMI, muscle attenuation, or BIA parameters precede deterioration in patient-reported outcomes and whether interventions implemented in response to these changes improve quality of life and treatment tolerance.

## 7. BIA as a Supportive Care Tool

Computed tomography, particularly cross-sectional analysis at the level of the third lumbar vertebra, is widely used for the quantitative assessment of muscle tissue in oncology research. It enables measurement of skeletal muscle area, calculation of the skeletal muscle index, and assessment of muscle attenuation, which is considered an indirect imaging marker of muscle quality [27].

CT does not, however, permit a complete clinical diagnosis of sarcopenia, which also requires assessment of muscle strength and, in severe cases, physical performance. CT-derived parameters primarily reflect muscle quantity and radiological quality. Therefore, in studies based exclusively on imaging, the terms “low muscle mass” and “low muscle attenuation” are more precise than equating these findings with a complete diagnosis of sarcopenia.

An important advantage of CT analysis is the possibility of using images obtained during routine diagnostic assessment, disease staging, and treatment response monitoring. Secondary analysis of previously acquired scans does not expose patients to additional ionizing radiation [27]. The limitations of this method include the need for appropriate software, the time required for tissue segmentation, staff training, and standardization of the anatomical level, Hounsfield unit ranges, and cut-off values. In addition, CT examinations are not always conducted at intervals that permit regular monitoring of dynamic body composition changes. Emerging composite approaches may provide a more comprehensive assessment of muscle quality by integrating CT-derived skeletal muscle radiodensity with markers of systemic nutritional and inflammatory status. The albumin–myosteatosis gauge (AMG), calculated by multiplying skeletal muscle radiodensity by serum albumin concentration, was independently associated with poorer overall and progression-free survival in patients with advanced malignancies treated with immune checkpoint inhibitors, suggesting that it may capture prognostic information not fully reflected by isolated CT-derived muscle measures [28,29,30]. Similar findings have been reported in colorectal cancer, where AMG showed superior prognostic performance compared with skeletal muscle radiodensity or albumin alone, and in lung cancer treated with immunotherapy, where AMG predicted overall survival despite myosteatosis alone not being statistically significant [28,29,30]. Although these findings support the potential added value of multimodal indices, their prognostic utility and optimal cut-off values require specific validation in ovarian, endometrial, and cervical cancers before routine clinical application.

In this context, BIA represents a complementary method for repeated assessment of selected body composition parameters. It is noninvasive, rapid, relatively easy to use in outpatient settings, and does not involve exposure to ionizing radiation. Depending on the device and algorithms used, it can estimate lean body mass, skeletal muscle mass, total body water, and the ratio of extracellular water to total body water.

Evidence concerning the agreement between BIA and CT parameters was provided by the study by Lee et al. conducted among healthy adults [31]. The authors demonstrated a correlation between BIA-estimated skeletal muscle mass and CT-assessed trunk muscle volume. The correlation coefficient was 0.73 among women and 0.80 among men. BIA parameters were also associated with functional measures of muscle performance, including handgrip strength [31].

BIA and CT assess different tissue characteristics. CT allows direct measurement of the muscle area at a defined anatomical level and evaluation of its attenuation. BIA estimates body composition indirectly from the resistance and reactance of tissues to an electrical current, as well as from equations specific to a given device and population. BIA does not assess muscle quality in terms of radiological attenuation and cannot replace CT analysis in this respect.

The reliability of BIA measurements depends substantially on adherence to a standardized examination protocol. The results may are affected by hydration status, recent food and fluid intake, physical activity, ambient temperature, body position, bladder emptying, and the interval since the most recent treatment cycle. Edema, ascites, and electrolyte disturbances are particularly important because they may result in inaccurate estimates of lean body mass and muscle mass.

Sbrignadello et al. indicated that BIA was useful as an accessible and repeatable method of body composition assessment, while emphasizing the need to standardize measurement conditions and further validate the algorithms used [22].

In longitudinal studies, the direction and rate of change observed in an individual patient has a potential for more information than a single BIA result. Reliable comparisons require measurements to be performed using the same device, according to the same protocol, and under conditions that are as similar as possible. Hydration status, the presence of ascites or edema, and the current stage of treatment should be considered whenever changes are interpreted. Even when these principles are followed, BIA results should be analyzed together with body weight, nutritional status, clinical symptoms, muscle strength, and physical performance.

On the basis of the available evidence, BIA should not currently be regarded as an independent screening method for diagnosing sarcopenia in patients with gynecological malignancies. Uniform and adequately validated cut-off values for this population are lacking, and the results may vary depending on the device and prediction equations used. Nevertheless, BIA may support the monitoring of body composition changes and help identify patients who require more detailed assessment of nutritional status and physical functioning.

From the perspective of supportive care, BIA measurements are useful as one component of a broader monitoring process. Oncology nurses and midwives, depending on the organization of care, their qualifications, and professional scope of practice, may participate in standardized measurements, documentation of changes, and identification of concerning symptoms. Their role may also include educating patients about appropriate nutrition, safe physical activity, and the importance of reporting symptoms that limit food intake or daily functioning.

Low muscle mass can be assessed using several complementary methods. CT analysis at the third lumbar vertebral level is most frequently used in oncology to calculate skeletal muscle area and skeletal muscle index, while CT-derived muscle attenuation provides additional information on radiological muscle quality. DXA and BIA estimate whole-body or appendicular lean tissue, whereas muscle-strength and physical-performance tests provide the functional information required for the clinical assessment of sarcopenia. A practical comparison of methods used to assess muscle quantity, muscle quality, and physical function, including their advantages, limitations, and suggested clinical roles, is provided in Table 2.

Body composition measurements may also have educational and communication value because they can help explain changes that are not evident from body weight and BMI. However, this information should be communicated in a manner that does not result in stigmatization, excessive focus on body weight, or the belief that the patient is responsible for adverse changes associated with disease and treatment.

## 8. Implications for the Design of Future Clinical Studies

The available evidence supports the inclusion of body composition assessment in future observational and interventional studies involving patients with gynecological malignancies. Existing meta-analyses indicate that low muscle mass and low muscle attenuation are primarily associated with poorer overall survival and an increased risk of selected treatment-related complications [2,5]. Interpretation is limited, however, by differences in definitions of sarcopenia, measurement techniques, cut-off values, study population characteristics, and evaluated outcomes.

Future studies should clearly distinguish between the clinical diagnosis of sarcopenia and low muscle mass assessed solely by imaging methods. Whenever possible, body composition analysis should be complemented by evaluations of muscle strength, physical performance, and nutritional status. Standardization is also needed with respect to CT and BIA measurement procedures, devices and algorithms, cut-off values, and examination conditions.

Longitudinal studies comprising assessments before treatment initiation, during therapy, after treatment completion, and during long-term follow-up will be particularly valuable. This approach will enable the direction and pace of body composition changes to be determined, including changes that may remain undetected during routine monitoring of body weight and BMI [16,17]. Measurement time points should be adapted to cancer type, treatment regimen, and the primary aim of the study.

Body composition assessment should be integrated with clinical endpoints, including OS, PFS, postoperative complications, dose-limiting toxicity, dose reductions, treatment delays or discontinuation, and unplanned hospitalizations. Parallel monitoring of patient-reported outcomes is also important, including quality of life assessed using the EORTC QLQ-C30 and appropriate cancer-specific modules. Advanced weight loss has been associated with poorer functioning and quality of life, but this association cannot automatically be extrapolated to changes in SMI or BIA parameters [25].

A key direction for further research is to determine whether the detection of adverse body composition changes and the implementation of targeted interventions improve clinical outcomes. The association between low muscle mass and poorer prognosis does not establish that increasing muscle mass will reduce toxicity or prolong survival. Interventional studies should therefore assess not only body composition changes but also muscle strength and physical performance, nutritional status, delivery of planned treatment, and quality of life [1].

## 9. Conclusions

Low muscle mass and low muscle attenuation are associated with poorer overall survival and a higher risk of selected postoperative complications among patients with gynecological malignancies. This association is best documented in ovarian cancer, whereas findings concerning progression-free survival, disease recurrence, and systemic treatment toxicity remain less conclusive. Interpretation of the available evidence is limited by differences in the definitions of sarcopenia, measurement methods, and cut-off values. Low muscle mass and low muscle attenuation are associated with poorer overall survival and a higher risk of selected postoperative complications in gynecological oncology, but the certainty and clinical context of this association differ by cancer type. The evidence is most extensive for ovarian cancer, whereas data for cervical and endometrial cancer are less abundant, more treatment-specific, and methodologically heterogeneous.

Body composition assessment provides information that cannot be obtained from body weight and BMI alone. However, imaging-defined low muscle mass should be distinguished from a clinical diagnosis of sarcopenia, which also requires the assessment of muscle strength and, in severe cases, physical performance. Body composition analysis should therefore complement, rather than replace, a comprehensive assessment of nutritional status, functioning, and the clinical course of disease.

Low muscle mass and a higher drug dose relative to lean body mass are associated with an increased risk of chemotherapy toxicity, including paclitaxel-induced peripheral neuropathy. These findings do not currently justify routine modification of cytotoxic drug doses on the basis of body composition parameters, but they support further evaluation of these parameters as potential markers of adverse-event risk.

CT remains the principal method for quantitatively and qualitatively assessing muscle tissue in oncology research, particularly when secondary analysis of images obtained during routine diagnostic assessment is possible. BIA may serve as a practical complementary tool for repeated monitoring of body composition changes, but it does not replace CT analysis or functional assessment. Its usefulness in patients with gynecological malignancies requires further validation, particularly in the presence of ascites, edema, and hydration disturbances.

Direct evidence concerning the association between body composition and quality of life in gynecological oncology remains limited. Measurements of muscle mass and quality should therefore be combined with assessments of physical performance, nutritional status, symptom burden, and patient-reported outcomes. Early identification of adverse changes may facilitate referral for individually tailored nutritional, physiotherapeutic, and prehabilitation interventions. The available evidence suggests that the benefits of such interventions may primarily involve improvements in strength, physical capacity, and nutritional status, even without a short-term increase in muscle mass.

Future studies should include repeated body composition measurements, standardization of CT and BIA methods, and simultaneous assessment of muscle strength, physical performance, treatment toxicity, and quality of life. Interventional studies will be essential to determine whether body composition monitoring and the implementation of targeted supportive care based on its results actually improve treatment tolerance and clinical outcomes among patients with gynecological malignancies.

## Figures and Tables

**Table 1:** Key studies evaluating low muscle mass, muscle attenuation, and sarcopenia in gynecological malignancies.

Study and Design	Cancer Setting	Exposure and Outcome	Reference Values	Main Findings
Lin et al. (2023) [3]; systematic review and meta-analysis; 16 studies, *n* = 2031	Gynecological malignancies	Imaging-defined sarcopenia; OS and PFS	Heterogeneous imaging-based definitions across included studies; predominantly study-specific CT-derived SMI cut-offs. No uniform threshold was applied.	OS: HR 2.61 (95% CI 1.52–4.46); PFS: HR 1.37 (95% CI 1.09–1.73).
Allanson et al. (2020) [14]; systematic review and meta-analysis; 13 studies, *n* = 2446	Gynecological malignancies	Sarcopenia; OS, PFS, and treatment-related adverse events	Heterogeneous definitions across included studies; most studies used CT-derived SMI, with study-specific thresholds. No uniform cut-off was applied.	OS: HR 1.33 (95% CI 1.08–1.64); PFS: HR 1.69 (95% CI 1.03–2.76); adverse events: HR 1.28 (95% CI 0.69–2.40), not statistically significant.
Ubachs et al. (2019) [2]; systematic review and meta-analysis	Ovarian cancer	Low SMI and low muscle attenuation; OS	Heterogeneous SMI and skeletal muscle radiodensity/attenuation thresholds across the included studies; no single pooled diagnostic cut-off.	Low SMI: HR 1.11 (95% CI 1.03–1.20); low muscle attenuation: HR 1.14 (95% CI 1.08–1.20). Considerable heterogeneity in cut-off values was noted.
Ge et al. (2023) [5]; systematic review and meta-analysis; 22 studies	Ovarian cancer	Low SMI and low muscle attenuation; survival, recurrence, and complications	Study-specific CT-derived SMI and muscle attenuation thresholds; substantial between-study heterogeneity and no uniform cut-off.	OS: HR 1.30 (95% CI 1.07–1.58) for low SMI and HR 1.24 (95% CI 1.03–1.49) for low attenuation. Complications: RR 1.49 (95% CI 1.13–1.98) and RR 1.99 (95% CI 1.04–3.79), respectively. PFS and recurrence associations were not significant.
Bronger et al. (2017) [7]; observational study	Advanced high-grade serous ovarian cancer	Low baseline muscle mass; OS	Low muscle mass defined using CT-derived SMI at L3; the study applied a published female-specific SMI threshold.	Higher mortality risk: HR 2.89 (95% CI 1.11–7.54).
Lee et al. (2018) [8]; longitudinal imaging study	Locally advanced cervical cancer treated with chemoradiotherapy	SMI loss >10% per 150 days; OS	Longitudinal muscle loss defined as an SMI decrease >10% per 150 days during chemoradiotherapy.	Higher mortality risk: HR 6.02 (95% CI 3.04–11.93).
de Paula et al. (2019) [9]; observational study	Endometrial cancer	Low SMI; 1-year survival	Low SMI defined as <38.9 cm^2^/m^2^; low- and high-radiodensity muscle subranges were −29 to +29 HU and +30 to +150 HU, respectively.	Poorer 1-year survival: HR 2.23 (95% CI 1.19–4.20).
Mercan et al. (2021) [10]; surgical cohort	High-grade serous ovarian cancer undergoing CRS and HIPEC	Low muscle mass; infectious complications	Sarcopenia defined using a sex-specific total psoas index threshold measured at L3 on preoperative CT.	Higher risk of infectious complications: RR 3.16 (95% CI 1.21–8.28).
van der Zanden et al. (2021) [11]; surgical cohort	Older women with ovarian cancer	Low SMI and low muscle attenuation; postoperative infections	Low SMI defined as <38.5 cm^2^/m^2^; low muscle attenuation defined as <22.55 HU.	Low SMI: RR 2.00 (95% CI 1.06–3.77); low attenuation: RR 2.18 (95% CI 1.26–3.76).
Chae et al. (2021) [12]; cohort study	Early-stage ovarian cancer	Sarcopenia; length of hospital stay	Low SMI defined as ≤38.7 cm^2^/m^2^ on CT at L3.	Hospital stay: 17 days with sarcopenia versus 12 days with normal muscle mass.
Rutten et al. (2017) [13]; surgical cohort	Ovarian cancer undergoing primary debulking surgery	Sarcopenia; length of hospital stay	Low muscle mass defined using CT-derived SMI at L3 and a published female-specific threshold.	Hospital stay: 15.3 versus 13.6 days in patients with and without sarcopenia, respectively.
Aichi et al. (2025) [21]; retrospective study, *n* = 98	Endometrial cancer treated with carboplatin-paclitaxel	Paclitaxel dose per kg LBM; CIPN	Paclitaxel exposure threshold of 8.12 mg/kg LBM for CIPN risk; body composition and LBM were assessed on pretreatment CT.	Independent association with CIPN: OR 2.58 (95% CI 1.08–6.19). At a cut-off of 8.12 mg/kg LBM, CIPN occurred in 52.0% versus 30.1% (*p* = 0.049).
Katayama et al. (2026) [23]; retrospective study, *n* = 20	Endometrial cancer treated with immune checkpoint inhibitors	Sarcopenia at ICI initiation and change in SMI between baseline before any cancer-directed therapy and ICI initiation; OS and PFS	Sarcopenia at ICI initiation defined as SMI < 38.5 cm^2^/m^2^; ΔSMI calculated between CT before any cancer-directed therapy and CT immediately before ICI initiation.	Decreased pretreatment SMI was associated with worse OS: HR 3.64 (95% CI 1.11–11.94). Sarcopenia at ICI initiation showed a trend toward poorer OS and PFS. Among nonsarcopenic patients, each 1-unit decrease in SMI was associated with worse OS: HR 1.64 (95% CI 1.05–2.63).
Lee et al. (2023) [24]; retrospective cohort study, *n* = 650	Ovarian cancer treated with primary debulking surgery and adjuvant platinum-based chemotherapy	Pretreatment prognostic nutritional index and SMI loss between pre- and post-treatment CT; OS	Low pretreatment PNI defined as <47.2; clinically relevant SMI loss defined as a reduction exceeding 4.2% between pre- and post-treatment CT.	Low pretreatment PNI was associated with SMI loss: OR 1.97 (*p* = 0.001). Low PNI and SMI loss were independently associated with mortality: HR 1.43 (*p* = 0.017) and HR 2.27 (*p* < 0.001), respectively. Patients with both low PNI and SMI loss had approximately threefold higher mortality: HR 3.10 (*p* < 0.001).

Abbreviations: BMI, body mass index; CI, confidence interval; CIPN, chemotherapy-induced peripheral neuropathy; CRS, cytoreductive surgery; CT, computed tomography; HIPEC, hyperthermic intraperitoneal chemotherapy; HR, hazard ratio; LBM, lean body mass; OR, odds ratio; OS, overall survival; PFS, progression-free survival; RR, risk ratio; SMI, skeletal muscle index. Effect estimates are reported as presented in the cited studies and should be interpreted in light of heterogeneity in definitions and cut-off values.

**Table 2:** Complementary components of multidimensional sarcopenia assessment and supportive-care monitoring in gynecological oncology.

Assessment Component	Primary Information	Advantages	Key Limitations	Suggested Role
Body weight and BMI	Overall body size and change in weight	Simple, inexpensive, and suitable for frequent monitoring	Cannot distinguish muscle, fat, and fluid compartments; normal or high BMI may mask low muscle mass	Initial context and longitudinal trend; should not be used as the sole screen for adverse body composition
CT-derived SMI at L3	Cross-sectional skeletal muscle area normalized to height	Uses routine oncological scans; quantitative; no additional radiation when existing images are analyzed	Requires segmentation software, trained staff, standardized anatomical level and cut-offs; scan timing may not match monitoring needs	Principal imaging measure of muscle quantity in oncology research and risk stratification
CT muscle attenuation	Muscle radiodensity in Hounsfield units, an indirect marker of intramuscular lipid infiltration	Adds information on muscle quality beyond muscle area	Affected by acquisition and reconstruction parameters; heterogeneous thresholds; does not measure strength	Complementary imaging marker, particularly when evaluating prognosis and postoperative risk
Bioelectrical impedance analysis	Estimated lean mass, skeletal muscle mass, total body water, and extracellular-to-total body water ratio, depending on the device	Noninvasive, rapid, radiation-free, and suitable for repeated outpatient measurements	Indirect and device-dependent; influenced by hydration, recent intake, activity, body position, edema, ascites, and electrolyte disturbances; no validated gynecologic-oncology cut-offs	Complementary longitudinal monitoring under standardized conditions; not a stand-alone diagnostic test for sarcopenia
Muscle strength	Functional force-generating capacity, commonly assessed by handgrip strength	Central to the contemporary clinical definition of probable sarcopenia; responsive to functional change	Affected by pain, neuropathy, fatigue, motivation, and technique; requires standardized procedure	Should accompany body composition assessment to avoid equating low muscle mass with clinical sarcopenia
Physical performance	Mobility and integrated physical function	Identifies severe functional impairment and supports rehabilitation planning	May be limited by acute illness, postoperative restrictions, pain, or neurological deficits	Adds severity and functional context; useful for monitoring prehabilitation and supportive care
Nutritional assessment and patient-reported outcomes	Nutritional risk, food intake, symptoms, fatigue, and health-related quality of life	Captures factors and consequences not visible on imaging or BIA; supports individualized referral	Subject to reporting and instrument-related limitations; weight loss cannot be equated directly with muscle loss	Integrates body composition with symptom burden, treatment tolerance, and quality of life (e.g., EORTC QLQ-C30)

Abbreviations: BIA, bioelectrical impedance analysis; BMI, body mass index; CT, computed tomography; EORTC QLQ-C30, European Organisation for Research and Treatment of Cancer Quality of Life Questionnaire-Core 30; L3, third lumbar vertebra; SMI, skeletal muscle index. No single component is sufficient for a complete clinical diagnosis of sarcopenia; findings should be interpreted together with clinical status and treatment context.

## Data Availability

No new data were created or analyzed in this study. Data sharing is not applicable to this article.
